# Solubility Optimization of Loxoprofen as a Nonsteroidal Anti-Inflammatory Drug: Statistical Modeling and Optimization

**DOI:** 10.3390/molecules27144357

**Published:** 2022-07-07

**Authors:** Mohammed Alqarni, Nader Ibrahim Namazi, Sameer Alshehri, Ibrahim A. Naguib, Amal M. Alsubaiyel, Kumar Venkatesan, Eman Mohamed Elmokadem, Mahboubeh Pishnamazi, Mohammed A. S. Abourehab

**Affiliations:** 1 Department of Pharmaceutical Chemistry, College of Pharmacy, Taif University, Taif 21944, Saudi Arabia; m.aalqarni@tu.edu.sa (M.A.); i.abdelaal@tu.edu.sa (I.A.N.); 2 Pharmaceutics and Pharmaceutical Technology Department, College of Pharmacy, Taibah University, Al Madinah Al Munawarah 30001, Saudi Arabia; Nnamazi@taibahu.edu.sa; 3 Department of Pharmaceutics and Industrial Pharmacy, College of Pharmacy, Taif University, Taif 21944, Saudi Arabia; s.alshehri@tu.edu.sa; 4 Department of Pharmaceutics, College of Pharmacy, Qassim University, Buraidah 52571, Saudi Arabia; alsubaiyela@gmail.com; 5 Department of Pharmaceutical Chemistry, College of Pharmacy, King Khalid University, Abha 62529, Saudi Arabia; kumarve@kku.edu.sa; 6 Department of Pharmacy Practice and Clinical Pharmacy, Faculty Pharmacy, Future University in Egypt, New Cario 11835, Egypt; eman.abdelatif@fue.edu.eg; 7 Institute of Research and Development, Duy Tan University, Da Nang 550000, Vietnam; 8 The Faculty of Pharmacy, Duy Tan University, Da Nang 550000, Vietnam; 9 Department of Pharmaceutics, Faculty of Pharmacy, Umm Al-Qura University, Makkah 21955, Saudi Arabia; maabourehab@uqu.edu.sa; 10 Department of Pharmaceutics and Industrial Pharmacy, Faculty of Pharmacy, Minia University, Minia 61519, Egypt

**Keywords:** solubility, machine learning, supercritical fluids, optimization, loxoprofen

## Abstract

Industrial-based application of supercritical CO_2_ (SCCO_2_) has emerged as a promising technology in numerous scientific fields due to offering brilliant advantages, such as simplicity of application, eco-friendliness, and high performance. Loxoprofen sodium (chemical formula C_15_H_18_O_3_) is known as an efficient nonsteroidal anti-inflammatory drug (NSAID), which has been long propounded as an effective alleviator for various painful disorders like musculoskeletal conditions. Although experimental research plays an important role in obtaining drug solubility in SCCO_2_, the emergence of operational disadvantages such as high cost and long-time process duration has motivated the researchers to develop mathematical models based on artificial intelligence (AI) to predict this important parameter. Three distinct models have been used on the data in this work, all of which were based on decision trees: K-nearest neighbors (KNN), NU support vector machine (NU-SVR), and Gaussian process regression (GPR). The data set has two input characteristics, P (pressure) and T (temperature), and a single output, Y = solubility. After implementing and fine-tuning to the hyperparameters of these ensemble models, their performance has been evaluated using a variety of measures. The R-squared scores of all three models are greater than 0.9, however, the RMSE error rates are 1.879 × 10^−4^, 7.814 × 10^−5^, and 1.664 × 10^−4^ for the KNN, NU-SVR, and GPR models, respectively. MAE metrics of 1.116 × 10^−4^, 6.197 × 10^−5^, and 8.777 × 10^−5^errors were also discovered for the KNN, NU-SVR, and GPR models, respectively. A study was also carried out to determine the best quantity of solubility, which can be referred to as the (x_1_ = 40.0, x_2_ = 338.0, Y = 1.27 × 10^−3^) vector.

## 1. Introduction

The invention of novel drugs and the development of promising therapeutic approaches can be considered as the most important challenges in the pharmaceutical industry [1,2]. Perceiving the solubility behavior of various therapeutic drugs is known as a vital key point toward the efficient designation of the supercritical approach for the pharmaceutical industry [3]. Solubility can be defined as the capability of a solute to be dissolved in a particular solvent to obtain a homogenous Loxoprofen sodium is known as an important nonsteroidal anti-inflammatory drug (NSAID), which has considerable analgesic influence (10–20 times greater than ketoprofen or naproxen) that made it an appropriate anti-inflammatory and antipyretic agent [4]. Additionally, this drug has shown its great potential of application to relieve the acute/chronic pain without having serious side effects for the gastrointestinal tract [5,6]. Loxoprofen is instantly metabolized through the trans-alcohol formation, acts as a non-selective inhibitor of cyclooxygenase after oral administration, and reaches its maximum plasma concentration in less than 1 h [7]. Table 1 lists the structure and characteristics of Loxoprofen.

Over the last two decades, industrial-based application of supercritical CO_2_ (SCCO_2_) has attracted raised attention around the world due to their significant superioriy over organic solvents, such as non-toxicity, environmental-friendliness, and excellent efficiency [10,11]. In the case of environmental apprehension, SCCO_2_ is known as a green solvent, which possesses the diffusivity of gas and the density of liquid in the supercritical state. These interesting characteristics have provided an excellent opportunity for the replacement of SCCO_2_ to chemical solvents [12].

SCCO_2_ has a great ability to handle an extensive range of complicated challenges in pharmaceutical industries. This cutting-edge liquid solvent has been of great interest as a reliable method for traditional unit operations related to the pharmaceutical production process [13]. Determination of the optimized value of drug solubility is of great importance in the pharmaceutical industry due to its effect on momentous parameters such as size, shape, structure, and morphology [10]. Moreover, from the economic point of view, the performance of the supercritical method substantially relies on the true vision of drug solubility by supercritical fluids [14,15]. Numerous experimental/theoretical scientific studies have been conducted to understand the properties of SCCO_2_ systems, especially intermolecular interactions in supercritical fluid solutions [16,17,18]. Additionally, more progression has taken place in the application of SCCO_2_ as an alternative solvent system for materials’ processing [19,20,21]. 

It is no surprise that machine learning (ML) has become a gripping tool to enter the scientific disciplines recently. Recently, we are experiencing a blasting of work that develops and applies ML to a variety of scientific fields and domains [22,23,24,25]. In this study, three models were suggested to predict the solubility output given in the dataset, including KNN, Gaussian process regression, and NU-SVR. We also used a genetic algorithm (GA) for hyper-parameter tuning of these models.

The support vector machine model, or SVM, is a key approach in ML for many disciplines for different data sizes. The SVM provides rapid and robust answers to regression tasks [26,27,28]. SVM-based learning algorithms are especially appropriate to problems requiring previously unknown data, and they can be used to simply refine the solution. There are several different varieties of SVR algorithms, including Linear-SVR, LS-SVR, C-SVR, Nu-SVR, and many more [29,30].

It is the core idea behind k-nearest neighbors (KNN) models that they employ a similarity of input attributes of data to make predictions using other data points that are the most similar to the first. To be precise, it holds all of the training datasets during the testing phase as well [31,32].

Furthermore, the Gaussian process model (GPR) was introduced as a useful nonparametric Bayesian model which can be used in detection and utilization. The key benefit of GPR is the ability to accomplish a trustworthy response for the model’s initial attributes. By exploiting a theoretically infinite calculation of initial data and enables calculation of the model complication through Bayesian inference, this model might depict a wide range of relations among initial attributes and result values [33,34,35,36].

As mentioned before, the novelty of this study is using GA with three different new models to optimize the configurations (hyper-parameters) of them in order to optimize and predict the drug solubility. One of the best algorithms for addressing simple single-objective problems is the genetic algorithm (GA). Furthermore, many scholars examine multi-objective problems using the framework of GAs as the major body. Han et al. [37] proposed the fitting and interpolation-based multi-objective GA. The algorithm produced final solutions that outperformed other multi-objective algorithms in terms of diversity and convergence. It can achieve significantly better diversity and convergence in final solutions than other good multi-objective algorithms.

## 2. Data Set

In this work, a small dataset with 32 data points has been applied. Y = solubility is the only output of the experimental dataset used in this investigation, which has two input characteristics (pressure and temperature) and is displayed in Table 2. In Table 3, to determine the linear correlation among characteristics, the Pearson correlation coefficient is used. 

## 3. Methodology

The method has been used in this research is that the research data have been tested using all three models. For each model, the hyper-parameters of these models are searched and optimized using a genetic algorithm. Then, the best model with the best combination of parameters is selected and presented as the final research model.

The genetic algorithm (GA) [38] is a metaheuristic search algorithm based on Darwinian Theory, whose principle is “survival of the fittest”, where each subsequent generation outperforms the preceding generation. The multi-objective optimization technique and search problem are supported by the genetic algorithm.

GAs are among the most notable techniques under EAs, which are guided by evolutionary theories of genetic choice. They also embrace Charles Darwin’s philosophy of survival of the fittest. However, because of its superior optimization practice, GA has been referred as a function optimizer. The method has been started by loading a group of solutions (chromosome). It consists of a general explanation of the problem in the bit vector class. Later, compute fitness for each chromosome using a fitness function appropriate for the situation. Based on this, the most appropriate chromosomes are added to the matching pool, where they are subject to crossover and mutation, resulting in a diverse collection of solutions (offspring). Mutation, Crossover, and Selection are the three types of operators in Genetic Algorithm Selection:

- Natural selection is a process that causes evolutionary changes in organisms. The objective of optimization in our search algorithm is the maximum error of training data. Minimizing maximum error lets us to have the best hyper-parameter combination for each model.
- Mutation is a unary operator that operates on one chromosome at a time.
- Crossover is a binary operator that can utilize two chromosomes at the same time.

### 3.1. K-Nearest Neighbors (KNN) 

It is the core concept underlying k-nearest neighbors (KNN) models that they employ the similarity of input attributes of data to make predictions using other data points that are the most similar to the first. To be precise, it holds all of the training dataset during the testing phase as well [31]. To use KNN regression, we just need to adjust how many nearest neighbors that have the same numerical values as we do [39]. Another aspect is to look at the data is to weigh the closest neighbors inversely based on how far they are from the center. When regression is used, the same distance functions is utilized as when KNN is an applied classification to figure out how far away the samples are from each other. The following equations show how the distance is calculated between *x* and *y*, which are the two input vectors:

(1) $$Euc\_Distance=\sqrt{\overset{k}{\underset{i=1}{\sum }}{\left(x_{i}-y_{i}\right)}^{2}}$$

(2) $$Man\_Distance=\overset{k}{\underset{i=1}{\sum }}\left|x_{i}-y_{i}\right|$$

KNN trains by comparison of a given test instance (*X*, *y*) to a training dataset $S=\left\{\left(X_{\mathrm{ind}},y_{\mathrm{ind}}\right)\right\}$ For example, KNN computes the *d_ind,_* which is distance between *X* and each sample *X_ind_* in *S* and arranges the distance *d_ind_* according to its value. Accordingly, if *d_ind_* is ranked *ind*, then the instance associated to *d_ind_* is referred to as the *ind-th* closest neighbor, and the result is shown as *y_ind_* (*X*). Lat prediction *y* refers to the average of its k closest neighbor’s outputs in regression, as illustrated in the following equation [40,41].

(3) $$\hat{y}=\frac{1}{k}\sum_{\mathrm{ind}=1}^{k}y_{\mathrm{ind}}\left(X\right)$$

### 3.2. NU-SVR

As a flavor of support vector machine regression models, Nu-SVR had shown significant performance in many case studies. As the basic assumption considers a set of input and output values, as shown in [42]:

(4) $$\left\{\left(x_{1},y_{1}\right),\;\ldots ,\left(x_{n},y_{n}\right)\right\}$$

The objective of the Nu-SVR model is to find a nonlinear correlation exhibited in the down equation, as *f*(*x*) which should be adjacent to y as it is possible. In addition, it should be as flat as feasible [43]:

*f(x)* = *w^T^ P(x)* + *b* (5)

In the mentioned equation, *b* indicates the bias and *P*(*x*) is a nonlinear function that shows the current initial area to an area with more demotions, and *w^T^* is the weight vector. Getting the determined function to achieve the two basic goals of closeness and flatness is the primary focus of the task. In fact, the primary aim of the task is to modify [43]:

(6) $$\frac{1}{2}{\left|\lceil w\rceil \right|}^{2}+C\left\{Y.ɛ+\frac{1}{n}\sum_{i=1}^{n}\left(\xi +\xi^{*}\right)\right\}$$

With the conditions below [43]:

(7) $$y_{i}-\langle w^{T}.P\left(x\right)\rangle -b\leq ɛ+\xi_{i}^{*}$$

(8) $$\langle w^{T}.P\left(x\right)\rangle +b-y_{i}\leq ɛ+\xi_{i}$$

(9) $$\xi_{i}^{*},\xi_{i}\geq 0$$

Here, ε denotes a disparity of the *f*(*x*) of the actual observed amount, and *ξ*, *ξ_i_* are very weak variables declared in [44], which shows the disparity of *ξ* amount above ε error are reasonable. 

### 3.3. Gaussian Process Regression (GPR)

Resilience to errors in learning may often be improved using probabilistic regression. On one of the nonlinear regression approaches that uses a probabilistic regression framework but does not use parametric models, is the Gaussian Process Regression (GPR) [45]. In this method, the result variable *y* can be provided as follows:

(10) y=f(x(k))+ε

*x* reflects a calculation of result data, *f* identifies the uncertain functional dependence, and ξ refers to Gaussian noise $\;\sigma_{n}^{2}$ is the variance of Gaussian noise [46].

The results value is calculated using the Gaussian distribution *p* (*y*_∗_|*X*, *y*, *x*_∗_) by [47]:

(11) $$\begin{array}{cc} & {\hat{y}}_{*}=m\left(x_{*}\right)+k_{*}^{T}{\left(K+\sigma_{n}^{2}I\right)}^{-1}\left(y-m\left(x_{*}\right)\right),\\ & \sigma_{y_{*}}^{2}=k_{*}+\sigma_{n}^{2}-k_{*}^{T}{\left(K+\sigma_{n}^{2}I\right)}^{-1}k_{*}, \end{array}$$

K refers to a covariance matrix via the elements *K_i,j_* = *cov*(*x_i_*, *x_j_*), vector *k*_∗_ by below equation [47]:

(12) [k*]i=cov(xi, x*) and k*=cov(x*, x*)

To make trustworthy predictions, the mean and covariance function attributes are computed applying the dataset. The attributes are illustrated as hyper-attributes due to the aspects of the predictive possible distribution. The hyper-attributes are basically formed through maximizing of log*p*(y|X) [48]:

(13) $$\log p\left(y|X\right)=-\frac{1}{2}y^{T}{\left(K+\sigma_{n}^{2}I\right)}^{-1}y-\frac{1}{2}\log\left(\left|K+\sigma_{n}^{2}I\right|\right)-\frac{n}{2}\log\left(2\pi \right)$$

where, *n* denotes the quantity of training subset.

## 4. Results

In order to test and analyze how well the provided models work in real data, final models will be built, and three metrics will be utilized to compare them. To find the optimal configurations of models, a genetic algorithm (GA) optimization was used. All implementations of this research have been used by applying Python programming language, which is a high-level language suitable for machine learning methods. This language is suitable for libraries, some of which include Sklearn, Numpy, Matplotlib, and Seaborn have been used in this research.

The horizontal area among two following amounts, especially the calculated and predicted results amount, can be calculated through the equation below; the mean absolute error (MAE) [49].

(14) $$MAE=\frac{1}{n}\sum_{i=1}^{n}\left|{\hat{y}}_{i}-y_{i}\right|$$

where *n* shows the amount of dataset and *y_i_* illustrates the real measured amount, and ${\hat{y}}_{i}$ shows the predicted amount.

A dataset’s standard deviation is measured utilizing the predicted amounts and the measured amounts are calculated via [49]:

(15) $$RMSE=\sqrt{\sum_{i=1}^{n}\frac{{\left({\hat{y}}_{i}-y_{i}\right)}^{2}}{n}}\;$$

(16) $$R^{2}=1-\frac{\sum_{i}{\left(y_{i}-{\hat{y}}_{i}\right)}^{2}}{\sum_{i}{\left(y_{i}-\mu \right)}^{2}}\;$$

*μ* shows the mean value of the real calculated values [49].

**Figure 1:**
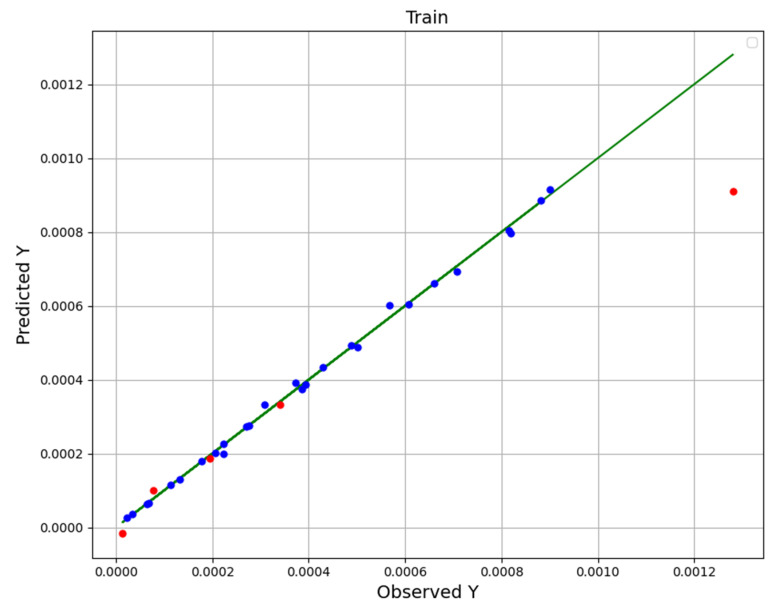
GPR fitting chart.

**Figure 2:**
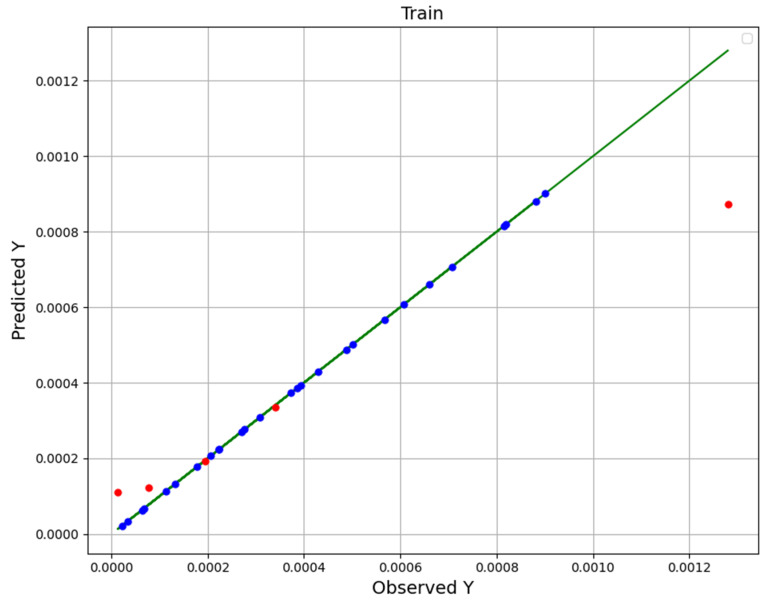
KNN fitting chart.

**Figure 3:**
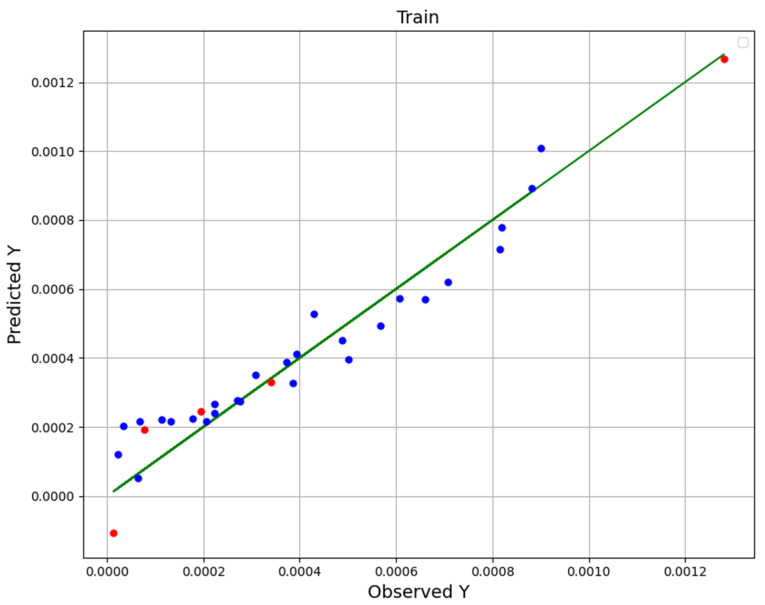
NU-SVR fitting chart.

Given that all three models have good performance and that the R-square of all three is higher than 0.9, we had a hard time choosing the most general model. For this purpose, we pay attention to Figure 1, Figure 2 and Figure 3, in which the real (observation) values (green line) are displayed with the predicted values (blue in the training and red in the test). However, the points in the NU-SVR model seem to be further away from the actual data line compared to the other two models. But considering that in the other two models there is at least one red dot (test) with a very large distance, NU-SVR is the most general model. In other words, NU-SVR is considered as a better model because all the test points have shown an average prediction error, but in two other models, many of the points have shown errors near to zero, although in other points the error is huge. Table 4 compares the MAE, R^2^, and RMSE values of all developed models.

To summarize, in addition to introducing the models and explaining how to tune their configuration (calibrate super-parameters), experimental data and model predictions are compared in the last three figures. The dots model predictions, and the line is experimental data. Therefore, the NU-SVR model is the final model (most accurate) which is selected in this study, and that is used to analyze the solubility of the drug, and the continuation of the results is based on this.

**Figure 4:**
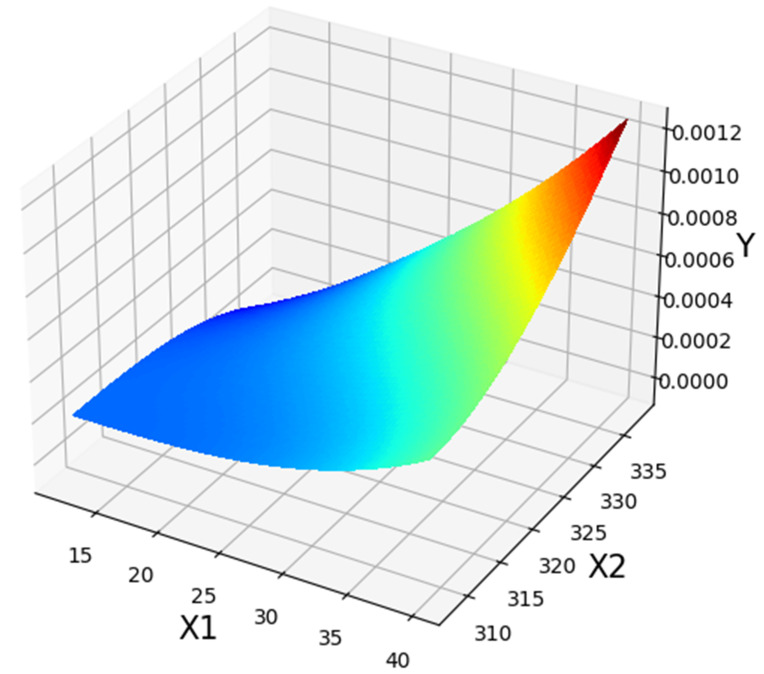
3D shows initial and result amounts (NuSVR Model).

**Figure 5:**
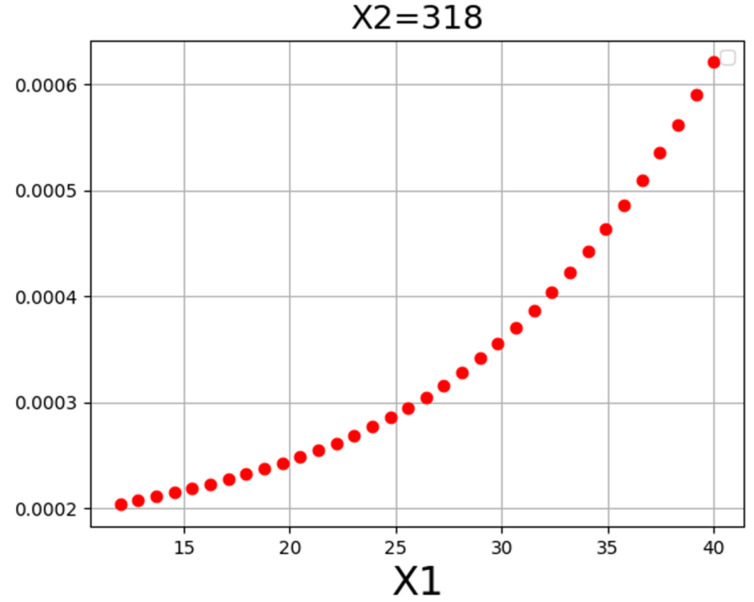
X1 tendency.

**Figure 6:**
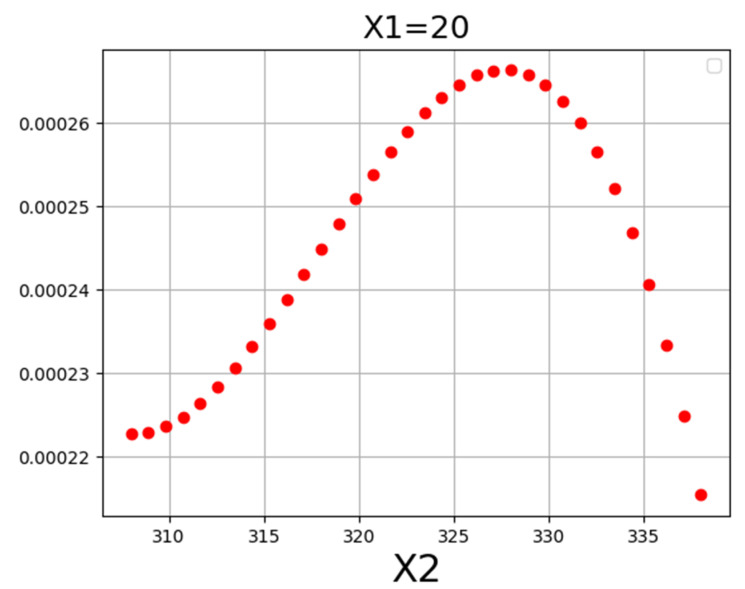
X2 tendency.

Figure 4 displays a 3D projection of the inputs onto a single output channel. It indicates that increasing the value of both traits will roughly increase output. Almost same fact is shown in Table 5 of optimal values. Detailed analysis in the demonstrated results of Figure 4, Figure 5 and Figure 6 imply the influence of pressure on drug solubility, directly. The increment of the pressure eventuates in improving the measured solubility amount of the drug. This problem may be justified because higher pressure positively encourages the density of SCCO_2_, which improves the solubilizing capability of SCCO_2_. Better speaking, by enhancement of pressure, the molecular arrangement occurs in a more compressed configuration, which changes the property of SCCO_2_ to a liquid-like fluid. By altering the property of the SCCO_2_ to a liquid-like fluid, its solvating strength significantly increases and, therefore, positively affects the solubility of the drug [50]. The second parameter is temperature, in which its variation has a significant impact on solubility. The impact of temperature on drug solubility is more complicated and needs more analysis. Temperature possesses a different influence on the density and pressure of the sublimation process, particularly when that is close to the solute melting point. Generally, an increase in temperature leads to an increment in molecular energy. Then, an increase in molecular energy results in higher propulsive forces and decreasing the density so that it causes a significant decrement in solvating of SCCO_2_. Density reduction results in the decrement of SCCO_2_ solvating followed by decreasing in the amount of Loxoprofen solubility in SCCO_2_. For pressures more than 27 MPa, temperature possesses reverse affection on drug solubility. It means that the pressure 27 MPa is identified as a turning amount, so that the affection of temperature on drug solubility is altered entirely. This attitude is due to the spontaneous impact of temperature on density decrement and the pressure of the sublimation process, which implies its indirect impact on the drug solubility in SCCO_2_. For the pressures below 27 MPa, the impact of density decrement because of temperature increase is more prevailing in comparison to the influence of the pressure of the sublimation process. If the amount is more than 27 MPa, the influence of temperature increment on the solid sublimation pressure is more than the density reduction, which causes the improvement of drug solubility. Following the abovementioned descriptions, pressure 27 MPa and temperature 338 K are obtained as optimized values for maximum response (1.268 × 10^−4^) hence considered optimized values. 

## 5. Conclusions

Obtaining the optimized solubility of various therapeutic drugs in SCCO_2_ in an extensive range of temperature and pressure is an attractive activity in the pharmaceutical industry. The main objective of this study is to employ three models based on AI technique to predict the optimized solubility of Loxoprofen sodium anti-inflammatory drug in SCCO_2_. For this purpose, three methods were used in this research to look at the data: KNN, NU-SVR, and Gaussian process regression. In this data set, there are two variables that can be changed: P (pressure) and T (temperature). The only output that can be obtained is Y, which is solubility. After setting up and fine-tuning the hyperparameters of these ensemble models, we looked at how well they did on several tests. This means that the RMSE error rates for the three models are all less than 0.99. The KNN, NU-SVR, and GPR models each have an RMSE error rate of 1.978 × 10^−4^, 7.814 × 10^−5^, and 1.660 × 10^−4^. This is not all: MAE metrics for the KNN model, NU-SVR model, and GPR model were also found to have 1.116 × 10^−4^, 6.197 × 10^−5^, and 8.777 × 10^−5^errors. A study was also undertaken to figure out the best amount of solubility, which can be described as (x_1_ = 40.0, x_2_ = 338.0, Y = 1.27 × 10^−3^) vector.

In a future work, we can use the same method for other drugs to obtain accurate models. The initial implementation has shown acceptable results for drugs such as Pholcodine, Ketoconazole, Galantamine, and lung in the same way.

## Figures and Tables

**Table 1:** Structure and characteristics of Loxoprofen [8,9].

Molecular Structure	Chemical Formula	Molecular Weight	Routes of Administration
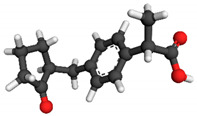	C_15_H_18_O_3_	246.306 g·mol^−1^	Oral, Transdermal

**Table 2:** The whole dataset.

No.	X1 = Pressure (MPa)	X2 = Temperature (K)	Y
1	12	308	6.75 × 10^−5^
2	16	308	1.32 × 10^−4^
3	20	308	1.77 × 10^−4^
4	24	308	2.23 × 10^−4^
5	28	308	2.77 × 10^−4^
6	32	308	3.41 × 10^−4^
7	36	308	3.93 × 10^−4^
8	40	308	4.29 × 10^−4^
9	12	318	3.46 × 10^−5^
10	16	318	1.13 × 10^−4^
11	20	318	1.94 × 10^−4^
12	24	318	2.70 × 10^−4^
13	28	318	3.85 × 10^−4^
14	32	318	5.01 × 10^−4^
15	36	318	5.67 × 10^−4^
16	40	318	7.07×10^−4^
17	12	328	2.20 × 10^−5^
18	16	328	7.65 × 10^−5^
19	20	328	2.23 × 10^−4^
20	24	328	3.09 × 10^−4^
21	28	328	4.88 × 10^−4^
22	32	328	6.60 × 10^−4^
23	36	328	8.16 × 10^−4^
24	40	328	8.81 × 10^−4^
25	12	338	1.35 × 10^−5^
26	16	338	6.33 × 10^−5^
27	20	338	2.07 × 10^−4^
28	24	338	3.73 × 10^−4^
29	28	338	6.07 × 10^−4^
30	32	338	8.20 × 10^−4^
31	36	338	9.01 × 10^−4^
32	40	338	1.28 × 10^−3^

**Table 3:** Pearson correlation analysis of data.

	X1	X2	Y
**X1**	1.0	−8.40 × 10^−16^	8.62 × −10^−1^
**X2**	−8.40 × 10^−16^	1.0	3.36 × −10^−1^
**Y**	8.62 × −10^−1^	3.36 × −10^−1^	1.0

**Table 4:** The outputs.

Models	MAE	R^2^	RMSE
**KNN**	1.116 × 10^−4^	0.917	1.879 × 10^−4^
**NU-SVR**	6.197 × 10^−5^	0.971	7.814 × 10^−5^
**GPR**	8.777 × 10^−5^	0.968	1.664 × 10^−4^

**Table 5:** Final attributes amount for the supreme outputs.

X_1_ = P (MPa)	X_2_ = T (K)	Y
40.0	338.0	1.268 × 10^−4^

## Data Availability

Not applicable.
